# Case report: Novel compound-heterozygous mutations in the *TCN2* gene identified in a chinese girl with transcobalamin deficiency

**DOI:** 10.3389/fgene.2022.951007

**Published:** 2022-08-12

**Authors:** Juan Luo, Hongxi Guo, Lifang Feng, Luhong Yang, Xiaoqian Chen, Tingting Du, Man Hu, Hui Yao, Xiaohong Chen

**Affiliations:** ^1^ Department of Endocrinology and Metabolism, Wuhan Children’s Hospital, Tongji Medical College, Huazhong University of Science and Technology, Wuhan, China; ^2^ Department of General Surgery, Wuhan Children’s Hospital, Tongji Medical College, Huazhong University of Science and Technology, Wuhan, China

**Keywords:** TCN2 gene, transcobalamin II (TC II) deficiency, vitamin B12 (cobalamin, VB12), megaloblastic anemia, whole-exome sequencing (WES)

## Abstract

Transcobalamin (TC) deficiency is a rare autosomal recessive disease characterized by megaloblastic anemia. It is caused by cellular vitamin B12 depletion, which subsequently results in elevated levels of homocysteine and methylmalonic acid. This disease is usually diagnosed by genetic analysis of the *TCN2* gene. Here, we described a 2.2-month-old Chinese girl with TC deficiency presenting with diarrhea, fever and poor feeding. Whole-exome sequencing detected a pair of compound-heterozygous mutations in *TCN2* gene, c.754-12C>G and c.1031_1032delGA (p.R344Tfs*20). To our knowledge, it is the first time that they were identified and reported in TC deficiency. This study contributes to a better understanding of the TC deficiency, expanding the spectrum of *TCN2* mutations in this disorder and also supporting the early diagnosis and proper treatment of similar cases in the future.

## 1 Introduction

Transcobalamin (TC) deficiency (MIM#275350), first described in 1971 (Hakami et al., 1971; Regec et al., 1995), is a rare autosomal recessive disorder (Watkins and Rosenblatt, 2011). *TCN2* gene is located on chromosome 22q12.2, spans 18 kb and contains nine coding exons. Homozygous or compound-heterozygous mutations of the *TCN2* gene are known to contribute to TC deficiency, including deletions, missenses and nonsenses (Arwert et al., 1986; Li et al., 1994; Regec et al., 1995; Watkins and Rosenblatt, 2020). TC is a transport protein responsible for transporting absorbed vitamin B12 (cobalamin, VB12) from the terminal ileum to the epithelial cells and facilitating its cellular uptake through receptor-mediated endocytosis (Nielsen et al., 2012). Deficiency of TC will eventually lead to a gradual depletion of intracellular cobalamin during the first week of life and secondary impairment of methionine synthetase and methylmalonyl-CoA mutase activities (Gherasim et al., 2013). Clinical presentations of TC deficiency include failure to thrive, diarrhea, megaloblastic anemia, pancytopenia, infections associated with hypogammaglobulinemia and immunodeficiency, and eventually neurologic abnormalities, especially when treatment is delayed. The diagnosis of TC deficiency is initially based on the combination of clinical signs and laboratory findings, and ultimately confirmed by genetic testing. Homocysteine and methylmalonic acid levels were also found to be elevated in patients with TC deficiency. Early recognition of this rare disease and initiation of parenteral VB12 therapy is critical to control the progression of the disease and improve the prognosis.

In this study, we report the clinical, laboratory, genetic findings and treatment regimen of a Chinese girl with TC deficiency, in particular a novel pair of compound-heterozygous variants in the *TCN2* gene that she carries, as identified by whole-exome sequencing (WES). We also highlight the importance of early diagnosis and proper treatment of TC deficiency.

## 2 Methods

Informed written consent was obtained from the patient’s parents. This study was approved by the Medical Ethics Committee of Wuhan Children’s Hospital (No. 2022R038).

### 2.1 Clinical data collection

Clinical information was collected from official electronic medical records and follow-up visits. Physical and biochemical examinations were performed on admission, which include a complete blood count, blood gas analysis, blood sugar test, coagulation test. The levels of serum homocysteine, VB12 and folate were also measured. Urinary organic acid was analysed by gas chromatography mass spectrometry. Blood amino acid and acylcarnitine profiling were performed by liquid chromatography-tandem mass spectrometry.

### 2.2 Whole-exome sequencing

Peripheral blood samples were obtained from the patient and her parents. WES was undertaken by Running Gene Inc (Beijing, China). Genomic DNA was isolated from peripheral blood using DNA Isolation Kit (Blood DNA Kit V2, CW2553). Concentrations were determined on a Qubit fluorometer (Invitrogen, Q33216) using Qubit dsDNA HS Assay Kit (Invitrogen, Q32851). Agarose gel (1%) electrophoresis was performed for quality control. DNA libraries were prepared with KAPA Library Preparation Kit (Kapa Biosystems, KR0453) following the manufacturer’s instructions. Purifications between steps were carried out with Agencourt AMPure XP beads. The libraries were estimated with Qubit dsDNA HS Assay kit (Invitrogen, Q32851). Hybridization of pooled libraries to the capture probes and removal of non-hybridized library molecules were carried out according to the SeqCap hybrid Mix system. Library molecules fished out by hybridization were carried out with Dynabeads® MyOne™ Streptavidin T1 (Invitrogen, 65,601). Sample dilution, flowcell loading and sequencing were performed according to the Illumina specifications. DNA libraries were sequenced on the Novaseq (Illumina, San Diego, CA, United States) with 150-bp paired ends. Quality control was applied to raw data (stored in FASTQ format), which was obtained from Novaseq to guarantee the meaningfulness of downstream analysis. The percentage of reads with average quality > Q30 and GC content distribution were calculated and summarized. High-quality paired-end reads were aligned to the human reference genome sequence from the UCSC database (GChR37hg19, http://genome.ucsc.edu/) using the Burrows-Wheeler-Alignment tool. All variants were filtered against ExAC (Lek et al., 2016), ESP6500 (Fu et al., 2013), ClinVar (Landrum et al., 2020),1,000 genomes project_EAS (Auton et al., 2015), Human Gene Mutation Database (HGMD). Obtained variants were further selected according to co-segregation. SNPs and indels occurring in exons and canonical splice sites were further analyzed. Selected variants were also classified based on American College of Medical Genetics and Genomics (ACMG) guidelines (Richards et al., 2015).

The candidate causal variants identified by WES were then confirmed by Sanger sequencing and pedigree analysis. Primers (Table 1) were designed using Primer Premier 5.0 (Premier Biosoft, United States), and PCR was carried out to amplify the fragments covering the mutated sites on the LifeECO Thermal Cycler TC-96/G/H(b)C (Bioer Technology Co., Ltd., China). PCR products were then purified and sequenced by the ABI 3730XL DNA Sequencer (Applied Biosystems, Thermo Fisher Scientific, United States). Sanger sequencing was shown by Chromas Lite v2.01 (Technelysium Pty Ltd., Tewantin, QLD, Australia).

**TABLE 1:** Primers of Sanger sequencing and reverse transcription PCR (rtPCR).

Primer	Sequence 5′->3′
c.754–12C>G Sanger sequencing forward primer	ATC​CAG​GCT​CTC​TGT​CCT​CA
c.754–12C>G Sanger sequencing reverse primer	GGG​AAC​CCT​CTC​CTC​TGT​TC
c.1031_1032delGA Sanger sequencing forward primer	GGC​ATT​ACA​GGT​GGG​AAA​GA
c.1031_1032delGA Sanger sequencing reverse primer	CAG​CAA​ATC​AGG​ATG​AAG​CA
rtPCR forward primer	AGG​ATG​GAG​CCT​TCC​AGA​AT
rtPCR reverse primer	AAG​CTG​CCA​GAA​CTC​CCT​TT

## 3 Results

### 3.1 Clinical presentation

A 2.2-month-old Chinese girl admitted to our hospital on the complaints of diarrhea, fever and poor feeding. She is the third child of her healthy non-consanguineous parents (G3P1). Previous pregnancies were spontaneous miscarriages. After an unremarkable pregnancy, she was born at 38-week gestation *via* vaginal delivery. Her birth weight was 3.05 kg (25–50th percentile), birth length was 49 cm (50th percentile), and head circumference 33 cm (50th percentile). No family history of associated diseases was reported, especially of the hematologic system.

#### 3.1.1 Physical examinations

The patient’s weight was 2.57 kg (<3rd percentile) and her length was 54 cm (3rd-10th percentile) at the age of 2.2 months. She developed severe malnutrition which manifested as marasmus. She had a less rosy complexion, pale lips, thin subcutaneous fat, poor skin stretch and petechiae. No abnormalities were found on her facial appearance, muscular tone or psychomotor development.

#### 3.1.2 Laboratory examinations

Complete blood count revealed that the patient had pancytopenia (Red Blood Cell Count (RBC) 2.25×10^12^/L (3.2–4.9), Hemoglobin (Hb) 73 g/L (90–140), White Blood Cell Count (WBC) 2.48×10^9^/L (5.0–12), platelets count 3×10^9^/L (100–378). Further biochemical examinations showed that she had an elevated urine methylmalonic acid level (23.87 μmol/L [0.2–3.6]) and an increased plasma homocysteine level (21.85 umol/l [<15]). The patient’s serum VB12 and folate levels were 275.60 pmol/L (139.4–651.5) and 17.86 nmol/L (7.0–46.4), respectively. Blood gas analysis revealed her metabolic acidosis. The girl had a slightly increased ratio of propionyl carnitine to acetylcarnitine (0.319 [0.04–0.25]). The results of other biochemical tests were normal.

### 3.2 Genetic analysis

**FIGURE 1:**
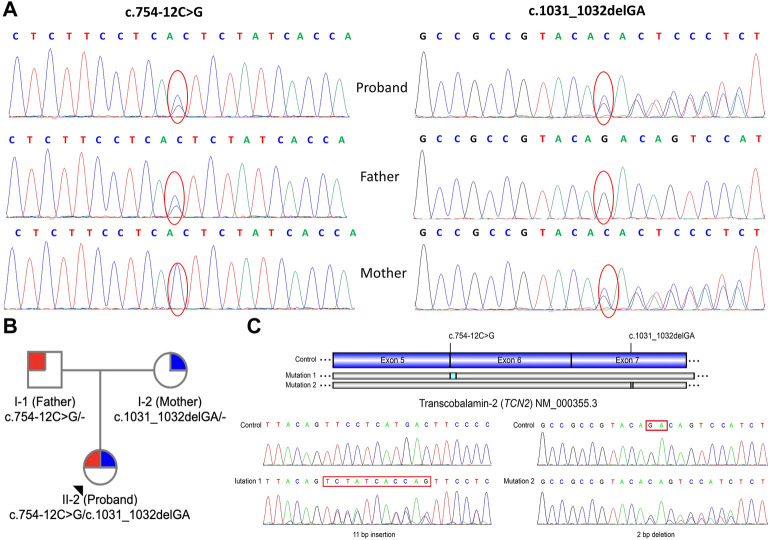
Genetic information of the patient. **(A)** Sanger sequencing showed a heterozygous maternally inherited variants (c.1031_1032delGA) and a heterozygous paternally inherited variant (c.754–12C>G) in *TCN2* gene in patient. **(B)** Pedigree analysis of this family. **(C)** Reverse transcription and Sanger sequencing of exon 3-7 of *TCN2* mRNA revealed an 11-base (TCTATCACCAG) insertion in the intron 5 and a 2-bp deletion (r.1031_1032delGA) in the exon 7.

To support the clinical diagnosis, WES was recommended and performed. A pair of compound-heterozygous variants, c.754-12C>G (intron 5) and c.1031_1032delGA (p.R344Tfs*20) (exon 7), in *TCN2* gene (NM_000355.3) were identified and subsequently validated by Sanger sequencing (Figure 1B).

Variant c.754-12C>G was absent from control databases (ExAC, 1000 Genomes Project, gnomAD and ESP6500) (PM2_supporting). Multiple *in silico* algorithms predicted that the variant may affect normal mRNA splicing (ADA, score = 0.9984 > cutoff = 0.6, damaging; RF, score = 0.96 > cutoff = 0.6, deleterious) (PP3). The patient’s symptoms are consistent with the specific manifestations of TC deficiency (PP4). Therefore, c.754-12C>G is classified as “variant of uncertain clinical significance” (PM2_supporting + PP3+PP4) on the basis of the ACMG guidelines.

Deletion c.1031_1032delGA results in a frameshift of the amino acid sequence (p.R344Tfs*20), which leads to a truncated protein (PVS1). This variant has not been reported in any public databases, including ExAC, 1000 Genomes Project, gnomAD and ESP6500 (PM2_supporting). The patient’s symptoms are consistent with the specific manifestations of TC deficiency (PP4). Thus, variant c.1031_1032delGA is classified as “pathogenic” (PVS1+PM2_Supporting + PP4), according to the ACMG guidelines.

Reverse transcription PCR and Sanger sequencing of exon 3-7 of *TCN2* mRNA revealed a 11-bp insertion (TCTATCACCAG) in the 5th intron (Figure 1C), supporting the prediction that variant c.754-12C>G affects mRNA splicing. A deletion (r.1031_1032delGA) was also identified in the exon 7, which is consistent with the mutation identified at the DNA level (c.1031_1032delGA). The study of the mRNA of *TCN2* gene enhanced the pathogenic potential of both novel variants we identified here.

### 3.3 Treatment and prognosis

Initially, patient received routine treatment including rehydration, correction of acidosis, and related symptomatic treatment. Antibiotics were also applied, but the symptoms did not improve. When the inborn error of cobalamin metabolism was suspected, hydroxocobalamin (1 mg) was given by intramuscular [i.m.] injection twice a week. All symptoms disappeared during her stay in hospital. Patient’s hematologic parameters, homocysteine levels and urine methylmalonic acid levels returned to the normal range (Table 2). At the patient’s last outpatient visit (2 years and 7 months old), neurological examinations revealed normal. Personal-social, fine motor-adaptive, language-speech and gross motor skills showed no significant differences compared with peers.

**TABLE 2:** Laboratory findings of patients before and after hydroxy-Cbl treatment.

Parameters	Before	After treatment				
Age	2 m2 d	2 m9 d	5 m	10 m	1 y7 m	2 y3 m
Hb (g/L) (90–140)	73	123	121	119	129	123
RBC (10^12^/L) (3.2–4.9)	2.25	4.41	3.98	4.32	4.61	4.55
MCV (fL) (75–121)	89.5	84.7	86.7	79.3	81.8	85.9
WBC (10^9^/L) (5–12)	2.48	9.85	9.79	8.2	7.35	4.8
ANC (10^9^/L) (1.08–5.9)	0.49	1.37	1.01	1.42	1.64	1.40
Plt (10^9^/L) (100–378)	3	334	261	242	237	144
Vitamin B12 (pg/ml) (139.4–651.5)	275.60	>1,400	>2000	>2000	>2000	-
Homocysteine (μmol/L) (<15)	21.85	6.71	13.1	5.36	5.60	8.93
Urine methylmalonic acid (μmol/L) (0.2–3.6)	23.87	3.20	1.54	0.92	1.14	1.11
Propionyl carnitine to acetylcarnitine (0.04–0.25)	0.319	0.10	0.04	0.05	0.06	0.14

Hb, hemoglobulin; RBC, red blood cell; MCV, mean corpuscular volume; WBC, white blood cell; ANC, absolute neutrophil count; plt, platelets.

## 4 Discussion

VB12 from food is bound to protein in the saliva, then it is digested by pancreatic enzymes. The released VB12 binds to the intrinsic factor until it reaches epithelial cells of terminal ileum, where VB12 dissociates from intrinsic factor, enters the portal circulation and binds to TC for cellular intake of VB12 by a process called micropinocytosis. Inside the consumers, cobalamin is then released and converted to methylcobalamin, a methyl donor for re-methylating homocysteine to methionine, and adenosylcobalamin, a cofactor converting methylmalonate to succinate (Rappazzo and Hall, 1972; Barshop et al., 1990; Banerjee and Ragsdale, 2003). Thus, VB12 plays an essential role in DNA synthesis and cellular metabolism (Hunt et al., 2014).

**FIGURE 2:**
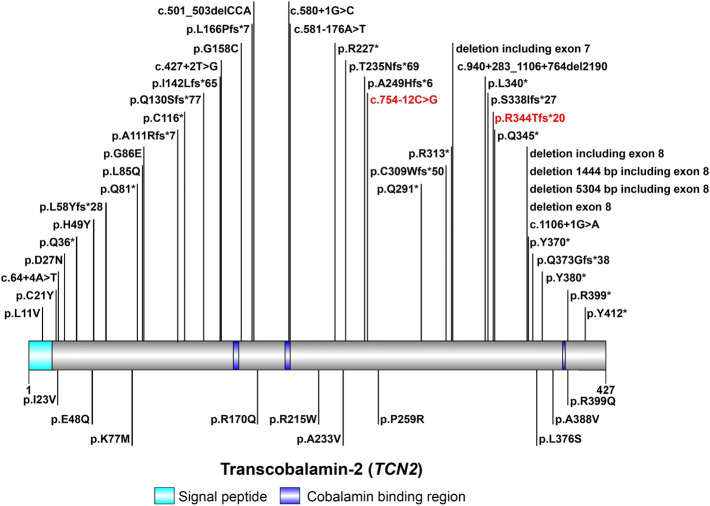
The spectrum landscape of mutations in *TCN2* gene. A total of 48 mutations and polymorphisms of *TCN2* gene were reported in HGMD. Disease-causing mutations (DMs) are marked on the top. Other non-DMs and polymorphisms are marked in the bottom. Two variants identified in this study are highlighted in red.

*TCN2* gene encodes TC to regulate the bioavailability of VB12 (Arwert et al., 1986; Regec et al., 1995; Zheng et al., 2017). The aberrant expression of *TCN2* gene is a mechanism contributing to the pathogenesis of TC deficiency. Currently, about 60 cases of TC deficiency have been reported worldwide (Trakadis et al., 2014; Kose et al., 2020), with only one case from China (Zhan et al., 2020). A total of 48 variants in *TCN2* gene have been reported in HGMD to date, including 38 disease-causing mutations (Figure 2). In patients with TC deficiency, deletions are the most common variant, with insertions and variants at splicing sites rarely found. The majority (about 80%) of patients carried homozygous variants, while only a minority had compound-heterozygous variants (Schiff et al., 2010; Trakadis et al., 2014; Bartakke et al., 2015; Chao et al., 2017; Li and Goubeaux, 2017; Yildirim et al., 2017; Ünal et al., 2019). In this study, a pair of compound-heterozygous variants in *TCN2* gene were identified, which is a very valuable case of TC deficiency.

TC deficiency may cause intracellular depletion of cobalamin, leading to functional folate deficiency and consequently leads to inhibition of DNA synthesis. It is therefore reasonable to infer that the lack of TC would lead to abnormalities in multiple systems, including hematologic, neurologic, immunological, gastrointestinal, dermal and reproductive systems. Trakadis et al. (2014) reported that 87.5% of patients showed hematological manifestations, including anemia and pancytopenia. Speech disorder is the most common neurological complication reported in the literature (Trakadis et al., 2014). Gastrointestinal complications are common in TC deficiency. A cohort study indicated that 37.5% patients presented with gastrointestinal symptoms, mainly vomiting and diarrhea, with a minority presenting with intestinal mucositis (Trakadis et al., 2014). The clinical presentations of pancytopenia, diarrhea, severe malnutrition and recurrent infections observed in our patient are consistent with previous reports.

TC deficiency is usually suspected and diagnosed based on the presence of clinical features and laboratory findings. A diagnosis of TC deficiency should be highly considered in any patient with megaloblastic anaemia who has elevated blood levels of VB12 and elevated levels of homocysteine and methylmalonic acid (Trakadis et al., 2014). However, it has been documented that a normal or low serum VB12 levels or normal homocysteine levels do not exclude TC deficiency (Ünal et al., 2019). Thus, the diagnosis of TC deficiency still needs to be confirmed by genetic analysis.

It is well known that early and proper treatment is crucial for achieving optimal outcomes (Yildirim et al., 2017; Ünal et al., 2019; Zhan et al., 2020). Most studies showed that early and aggressive treatment, which includes parenteral or high-dose (1 mg) i. m. (at least once a week), led to better outcomes (Yildirim et al., 2017; Ünal et al., 2019). Moreover, neurological and hematological deteriorations have been reported in patients who discontinued treatment (Yildirim et al., 2017; Ünal et al., 2019). In our patient, we adopted hydroxocobalamin (1 mg, i. m., twice a week) regimens. At clinical follow-ups, we found that the doses of medication used administrated to her were sufficient to restore her neurological and hematological parameters to normal levels.

In summary, the onset of TC deficiency often occurs early in life with multisystem involvement. Diagnostic workups, particularly hematological evaluations, can be misleading. Misdiagnosis and delayed proper treatment may result in permanent intellectual defects, blindness and motor abnormalities (Ünal et al., 2019). Once the diagnosis has been verified by genetic analysis of the *TCN2* gene, cyanocobalamin should be administered intramuscularly to the patient on a regular basis. The experience of this case will provide a practical basis for follow-up research. In particular, we identified two novel variants, c.754-12C>G and c.1031_1032delGA, expending the disease-causing mutation spectrum of *TCN2* gene.

## Data Availability

The datasets presented in this study can be found in online repositories. The names of the repository/repositories and accession number(s) can be found in the article/Supplementary Material.
